# Annotations

**Published:** 1909-05-01

**Authors:** 


### ANNOTATIONS.

### Reconciliation and Co-operation.

We have always believed that the wide differences of opinion which separate ourselves and the great body of medical and scientific men from the conscientious humanitarians need not prevent these two bodies of civilised persons from coming to some sort of terms, or at least uniting in a common campaign against wanton cruelty. There are immense numbers of educated and thoughtful persons, who differ from us upon the ethics and practical value of animal experimentation, but with whom we are willing to co-operate in the furtherance of humane objects which we as well as they have at heart. Quite lately we have had practical evidence of this spirit of mutual forbearance and common aspiration, and we feel that it is our duty to publish an outline of the circumstances. Many of us still hope to convince some at least amongst the earnest and sincere anti-vivisectionists, not only that vivisection is not as black as it is painted, but, that, as practised in England at the present day, it is not black at all, but far otherwise. And we have little doubt that the more tolerant and open-minded members of the humanitarian party live in hopes, for their part, of convincing us of the useless and retrogressive immorality of animal experimentation, even if we could prove to them that (as is almost exclusively the case) the researches are conducted under proper and complete anæsthesia, and with careful regard for the animals' feelings both before and after the experiments. Upon this ground we fear that we shall never agree; but we are glad to recognise that in fighting an abominable practice humanitarians and ourselves are willing and able to join forces. A few weeks ago we wrote confidentially to the Humanitarian League, drawing the attention of that society to the gross and nauseating barbarity of the method of skinning large snakes for commercial purposes which still exists in certain islands, happily not part of the British Empire. Our information came from a translation in *The Literary Digest*, and in his courteous reply to our letter Mr. Henry Salt, the Hon. Secretary of the League, concludes in the following terms, which to us are full of encouragement and promise: "I can assure you that we cordially welcome your kind co-operation, and that while we of course differ from you and your friends as to the wisdom of certain methods of research, we are quite aware that wanton cruelty is a wholly different matter, on which you feel as we do."

### Hygiene and Christian Science.

The proceedings at an inquest held last week upon the body of a lady who died at Sutton whilst residing as a patient with a household of Christian Scientists (self-styled) have been reported at some length in the daily press. There is little to distinguish this case from many others which have been made public in a similar way during recent years, unless it is that the "treatment" had less to do than is usual with the fatal issue, though it seems fairly clear that the unfortunate Miss Latour would have lived longer and suffered less had she never adopted the tenets of this peculiar sect. In commenting on these sad cases, there is always a tendency to regard exclusively the patient, and his or her individual disease and its neglect. But there is frequently a much wider aspect of matters, that in which the public health is concerned. The particular patient in the present instance was a sufferer from chronic pulmonary tuberculosis. She was treated at Kew by one of the practitioners who gave evidence at the inquest, and when he last attended her, some fifteen months before her death, the disease was entirely latent. Afterwards the tuberculosis evidently became once more active, with the result that the case came before the coroner. During this active period the patient was no doubt a source of grave danger to all who came into intimate contact with her; for, having been converted to Christian Science, she refused to have medical attendance. If the contention is to be admitted that there is no such thing as pain or disease, we presume it follows that there is no such thing as infection, and that prophylactic hygiene is useless. We have no information whether this is so, or whether the prevention of contagion is considered a suitable subject of prayer by Christian Scientists; but at any rate the significant fact emerges that the younger sister of the deceased, who lived in the same house until a few days before the latter's death, is now suffering from tuberculosis and has been removed to a sanatorium. When all is said and done, it is to the education of the public in hygiene that we must chiefly trust for the discrediting of these amateur therapeutists. There are at least twelve Sutton householders who, to judge by their verdict, are convinced that true science would have done better for Miss Latour than faith-healing did; and they have had a practical lesson in the tuberculosis problem which it is to be hoped they, and the rest of the public, will take seriously to heart.

### The Declining Cabman.

The appeal which Lord Rosebery has made on behalf of the London cabman, and with which the *Daily Mail* has so promptly and generously associated itself, is one which, we feel sure, will be cordially endorsed by every member of the medical profession. In the past the cabby has proved a good friend to the London doctor, and the present distress that threatens him is due to no fault of his own, but entirely to the altered conditions produced by the advent of the motor-taxicab. Practitioners and consultants cannot be blamed for using the more up-to-date and expeditious mode of conveyance in preference to the slower and more old-fashioned hansom, but they, above all, will recognise the gravity of the cabman's case, and join in the attempt to improve it by supporting a fund whose objects are to teach the younger men to become motor-drivers and to make provision for the immediate need of the older cabmen who, through age or for other reasons, may be compelled to seek another means of livelihood. Contributions towards the fund may be forwarded direct to the *Daily Mail*, which has undertaken the secretarial and executive work in connection with the appeal.

